# Pharmacokinetics and tissue distribution of LN002, a new compound alternative oxidase inhibitor against *Cryptosporidium* in rats

**DOI:** 10.3389/fphar.2024.1413872

**Published:** 2024-07-30

**Authors:** Minglang Ma, Yongxiang Zhang, Yanjun Fang, Yixing Lu, Huiguo Huang, Zhenling Zeng, Dongping Zeng

**Affiliations:** ^1^ Guangdong Provincial Key Laboratory of Veterinary Pharmaceutics Development and Safety. Evaluation, National Risk Assessment Laboratory for Antimicrobial Resistance of Animal. Original Bacteria, College of Veterinary Medicine, South China Agricultural University, Guangzhou, China; ^2^ Guangdong Laboratory for Lingnan Modern Agriculture, Guangzhou, China

**Keywords:** cryptosporidiosis, rat, alternative oxidase inhibitors, HPLC, pharmacokinetics, tissue distribution

## Abstract

Cryptosporidiosis is considered a crucial zoonotic disease caused by widely distributing parasitic protozoa called *Cryptosporidium* spp. Nitazoxanide is the only FDA-approved drug but is only effective with a good immune response of the host. In addressing this unmet medical need, we previously identified a compound, namely, LN002, as a potent alternative oxidase inhibitor against cryptosporidiosis. To illustrate the pharmacokinetics, absolute bioavailability, and tissue distribution of LN002 in rats, rapid and sensitive high-performance liquid chromatography was developed and validated for the separation and detection of LN002 in plasma, tissue samples, and intestinal contents. In this study, a single dose of oral administration and intravenous injection of LN002 was used to determine the levels of LN002 in plasma, tissue samples, and intestinal contents by UHLC. Results of the study indicated that after intravenous administration of 1 mg/kg LN002, the AUC0–24 h, T_1/2_,V_d_, and Cl were 7024.86 h·ng/mL, 10.91 h, 1.69 L/kg, and 0.11 L/h/kg, respectively. After oral administration of a single dosage of 100, 200, and 400 mg/kg LN002, the T_max_, C_max_, AUC_0–24 h_, T_1/2_, F, V_d_, and Cl/F in plasma of rats were 1 h, 849.88–4033.21 ng/mL, 2280.41–7498.10 h·ng/mL, 17.96–18.83 h, 0.27%–0.32%, 581.54–869.21 L/kg, and 25.97–39.00 L/h/kg, respectively. After oral administration of 200 mg/kg, LN002 was extensively distributed in the main tissues of rats, and massive amounts of LN002 were distributed in the intestine and intestinal contents, indicating its potential as an effective anti-Cryptosporidium compound. After oral administration of a single dosage of 200 mg/kg, LN002 has a low bioavailability and high levels in the intestine, which is crucial for the safe and effective treatment of cryptosporidiosis. Overall, the results of this study provide valuable data support for the future study of LN002.

## 1 Introduction

*Cryptosporidium parvum* is an obligate parasite of the phylum Apicomplexa that infects the microvilli of the small intestine of many mammalian hosts, including humans (Marzook and Sateriale, 2020), and causes self-limiting watery diarrhea or persistent and severe diarrhea called cryptosporidiosis depending on the age and immune status of the affected animals (Sonzogni-Desautels et al., 2015; Manjunatha et al., 2017; Schubert et al., 2023). Cryptosporidiosis is a zoonotic disease mainly transmitted through intake of food and water contaminated by *Cryptosporidium* (Ryan et al., 2016; Ryan et al., 2021). Given the increasing veterinary services and labor costs, increasing animal healthcare cost (Rahman et al., 2022a), and decreasing the growth rate of animals and mortality of severe animals (Shaw et al., 2020), cryptosporidiosis in livestock is becoming the remarkable problem for animal health (subclinical and clinical) and economic losses (Jacobson et al., 2016; Rahman et al., 2022b).

At present, nitazoxanide stands as the singular therapeutic agent ratified by the United States Food and Drug Administration for the explicit purpose of countering infections precipitated by *Cryptosporidium*, which has shown moderate efficacy (Tran et al., 2022; Khan and Witola, 2023; Yang et al., 2023). In addition, nitazoxanide is not effective without a good immune response of the host; thus, it is not used widely (Gargala, 2008). Nitazoxanide has been found to be ineffective in other immunodeficient or immunocompromised animal models of cryptosporidiosis, questioning the true efficacy of the drug (Lee et al., 2017; Jumani et al., 2018). Moreover, no effective therapeutic drugs are available for treating severe potentially life-threatening cryptosporidiosis in neonatal livestock. Accordingly, developing secure, cost-effective, and potent drugs is necessary to reduce the ever-increasing global cryptosporidiosis burden in the livestock sector, with a marked importance in resource-constrained nations.

*Cryptosporidium* has a single-host life cycle in which processes occur in the intestine of infected hosts, has adapted to the intracellular environment, has degeneration mitochondria remnants (powerhouses of the cell), and has aerobic and anaerobic metabolic pathways that are largely glycolytic (Zhang et al., 2012; Bouzid et al., 2013; Tandel et al., 2019). In *Cryptosporidium*, this glycolytic pathway is dependent on an additional step involving a protein called “alternative oxidase” (AOX). This protein is also present in microsporidia, but the absence of the protein in mammals, including humans, renders it a potential therapeutic target for the treatment of microsporidiosis (Williams et al., 2010; Sendra et al., 2022).

**FIGURE 1:**
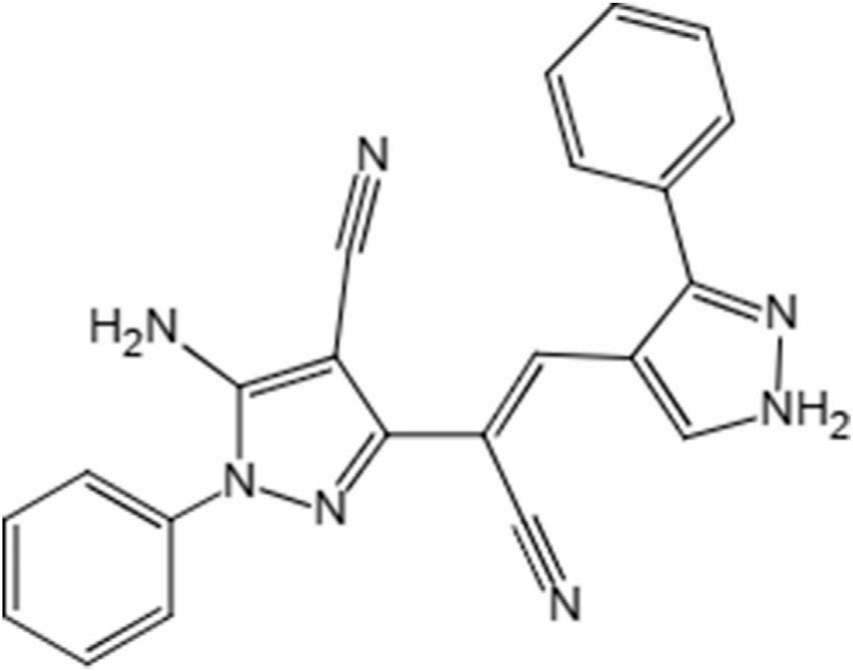
LN002 structural formula.

The compound 5-amino-3-[1-cyano-2-(3-phenyl-4-vinopyrazole))-1-phenyl-4-vinopyrazole (LN002) inhibits AOX for the treatment of cryptosporidiosis. It belongs to the family of phenylpyrazole, and it is structurally similar to fipronil with a chemical formula C22H15N7, and its molecular mass is 437.15 g/mol, Figure 1. Fipronil is a broad-spectrum phenylpyrazole pesticide and is used as an agricultural pesticide and as an ectoparasiticide in veterinary medicine (Roques et al., 2012; Ziliotto et al., 2017). Structurally, LN002 is made of two benzopyrazoles joined by carbon–carbon double bonds, and it does not contain any other halogen groups, making it less water soluble than fipronil. Therefore, LN002 may also be difficult to absorb in plasma. Considering that the infection site of *Cryptosporidium* is small intestinal mucosal epithelial cells, the compound may be more effective in the treatment of cryptosporidiosis if it is not absorbed in plasma. In previous studies, the compound LN002 was found to be effective and less cytotoxic in mice, but the oral absorption and distribution of LN002 in plasma, tissues, and intestinal contents remain unclear. Determining the absorption and distribution of LN002 in animals is helpful for further studies.

This study aimed to develop a valid method using liquid high-performance liquid chromatography (HPLC) to investigate the pharmacokinetics (PK) and tissue distribution of LN002 subsequent to administration. The study provides a methodology for studying the LN002 and understands the characteristics in pharmacokinetic and tissue distribution.

## 2 Materials and methods

### 2.1 Chemicals and reagents

LN002 (98%) was provided by Tianjin Ruipu Biotechnology Co., Ltd. (Tianjin, China). DMF was purchased from Damao Chemical Reagent Factory (Tianjin, China). Sodium carboxymethylcellulose was purchased from Zhongchen Biotechnology Co., Ltd (Henan, China). Chromatographicgrade methanol and methanol were purchased from Thermo Fisher Technology (Shanghai, China). Deionized water was prepared using a Milli-Q purification system (Millipore Corp., Bedford, MA, United States). Pentobarbital sodium and heparin sodium were obtained from Isejiu Biotechnology LLC (Jiangsu, China).

### 2.2 Experimental animals

The animal experimental protocol was reviewed and approved by the institutional animal care and use committee (approval number: 2023b101) by the Institutional Animal Experimentation Committee of South China Agricultural University and was consistent with the guidelines of the National Research Council. Male and female Sprague–Dawley (SD) rats (200–250 g, 7 weeks) were obtained from the Guangzhou Lingfu Tuopu Biological Co., Ltd. (Guangzhou, China). The animals were acclimated under a controlled environment with 12 h light/dark cycle for 5 days at 20°C–26°C and relative humidity of 40%–70%. The rats were housed in cages with toys, five rats per cage, and fasted for 12 h before the experiment, with free access to water and laboratory rodent diet (Guangzhou, China) (Tian et al., 2021).

### 2.3 Animal study

#### 2.3.1 Pharmacokinetics of LN002 in rat plasma content

A total of 24 SD rats, half for each sex, were randomly distributed into four groups and used in the plasma PK study. Heparin sodium was used to prevent blood clotting. Groups 1, 2, and 3 were single-dose oral administration groups, and the administered doses were 100, 200, and 400 mg/kg, respectively. The fourth group was a single-dose group, in which the subjects received 1 mg/kg LN002 via tail vein intravenous injection (In the oral administration group, the drugs were dissolved in 0.5% sodium carboxymethylcellulose, whereas those in the intravenous injection group were dissolved in N,N-dimethylformamide). After administration for each group, approximately 200–300 μL of plasma samples were collected from the tail vein at predefined times (0.167, 0.5, 0.75, 1, 2, 3, 4, 6, 8, 12, and 24 h) (Song et al., 2021a). Subsequently, these samples were then placed in centrifuge tubes containing heparin sodium, subjected to centrifugation at 5000 rpm for 10 min at 4°C, and the resulting supernatant was collected and stored at −20°C until further analysis.

#### 2.3.2 Tissue distribution

Thirty SD rats (15 for each sex) were randomly and equally assigned to 5 groups (half male and half female). The rats in each group were euthanized with pentobarbital sodium (a dose of 180 mg/kg) at five different time points: 0.5, 2, 6, 12 and 24 h after oral administration at 200 mg/kg LN002. Subsequently, various tissues, including the heart, liver, spleen, lungs, kidneys, brain, muscles, small intestine, and intestinal contents, were promptly collected (Verma et al., 2022). The dissected tissues were rinsed with normal saline, dried with an absorbent paper, homogenized using a tissue homogenizer, and stored at a temperature of −20°C until further analysis.

### 2.4 Standard solution and sample preparation

#### 2.4.1 Stock solution preparation

LN002 was dissolved in N,N-dimethylformamide, then mixed to make a concentration of 1 mg/mL standard solution, and stored at −20°C.

#### 2.4.2 Plasma and tissue and intestinal preparation

An aliquot of 100 µL of rat plasma was transferred into a 2 mL Eppendorf (EP) tube and precipitated with 300 µL of acetonitrile. The mixture was vortexed for 1 min, then centrifuged at 13,000 rpm at 4°C for 10 min, and passed through a 0.22 μm filter membrane. Final analysis was conducted by injecting 20 µL of supernatant into HPLC.

Tissue samples and intestinal contents were accurately weighed to 0.5 ± 0.02 g in a 10 mL EP tube and added with 2 mL of extraction solvent (acetonitrile: methanol [2:1, v/v]). The extraction solvent was vortexed for 1 min and centrifuged at 6,000 rpm and 4°C for 10 min. The supernatant was transferred to a new test tube and vortexed after adding 2 mL of water. The sample was purified using solid-phase extraction (SPE) using methanol as the extraction solvent. The Poly-Sery MAX SPE Cartridge (Shanghai Anpu Experimental Technology Co., Ltd., Shanghai, China) was added with 3 mL of methanol to activate, 3 mL of water to rinse, and the abovementioned extraction solvent. Afterward, 2 mL of 10% methanol water was poured into extraction columns to elute impurities. Finally, 1.5 mL of methanol was added to elute the compound and collect the eluent. Twenty microliters of the eluent was passed through a 0.22 μm filter membrane and then injected into the HPLC system for analysis.

### 2.5 Instrument and analytical conditions

A Shimadzu LC-20A system (Shimadzu Technologies, Kyoto, Japan) equipped with a LabSolutions system (version 5.1) system was used for HPLC analysis. The separation process utilized an ARD-C18 column (250 mm × 4.6 mm, 5 µm) (Zhongpu Technology Co., Ltd., Fuzhou, China). The analytes were detected using a UV detector at 333 nm. The chromatographic conditions used an isocratic mobile phase that combines methanol (solvent A) and deionized water (solvent B) (75:25, v/v), and they were propelled through the column at a constant flow rate of 1 mL/min, ensuring uniform separation. The injection volume was 20 μL. The temperature of the column was set to 40°C.

### 2.6 Method validation

The HPLC method was fully validated in accordance with Guidance for Industry Preparation of Veterinary New Drug submissions (Walker, 2018). The specificity, linearity range, limit of detection (LOD), limit of quantification (LOQ), intra-assay/inter-assay accuracy, precision, and stability under several conditions were determined.

#### 2.6.1 Specificity

To investigate whether the detection of LN002 could be affected by endogenous components in rat plasma, blank tissues, and intestinal contents, six blank plasma and blank tissues and intestinal contents were used. Using two distinct sets of blank plasma, tissue homogenates, and intestinal contents samples, one consists of blank samples, the other sample set was supplemented with LN002. The method’s exclusivity in detecting LN002 is confirmed through comparative analysis of the chromatograms.

#### 2.6.2 Linearity, LOD, and LOQ

Calibration curves were prepared by adding the working standard solutions into blank rat plasma to obtain calibration concentrations of 20, 50, 100, 200, 500, and 1,000 ng/mL; in the blank rat heart, liver, spleen, lungs, kidneys, brain, muscles, and small intestine homogenates and contents, the calibration concentrations were in the range of 50–2000 ng/g. Linearity was evaluated by the correlation coefficient (*R* ^*2*^), and a value of at least 0.99 was considered to be acceptable. Three replications of the calibration curve were performed on the same day (intraday).

#### 2.6.3 Accuracy and precision

The recovery was calculated by using two sets of samples. LN002 was evaluated at 20, 50, 200, and 1,000 ng/mL in blank plasma samples and 50, 200, and 1,000 ng/g in blank tissues (including the heart, liver, spleen, lungs, kidneys, brain, muscles, and small intestine and intestinal contents). The standard solution was diluted into a series of concentrations of LN002 working standard solutions for later use. For set 1, blank plasma, tissue homogenate, or intestinal contents were processed as described under sample preparation without a drug, followed by the addition of the abovementioned working solution, giving postextraction LN002 samples. For set 2, working solution and blank plasma, tissue homogenate, or intestinal contents were mixed and subjected to sample preparation, obtaining pre-extraction LN002 samples. Then, all samples were injected into the HPLC system for analysis. The recovery was quantified as the peak area ratio of the pre-extraction samples (set 2) to that of the postextraction samples (set 1).

#### 2.6.4 Stability studies

By investigating the low, middle, and high-concentration stability of rat plasma, the liver and intestinal contents in six replicates at different storage conditions, autosampler, short-term, freeze and thaw, long-term stability, and stock solution stability evaluations were conducted.

### 2.7 Data analysis

Pharmacokinetic data were obtained via non-compartmental PK analysis of total concentration *versus* time profiles for plasma using Phoenix WinNonlin 8.2 (Certara, L.P., Princeton, NJ, United States). Statistical analysis and graphing were undertaken using GraphPad Prism 9.5 for Windows (GraphPad Software, Inc., La Jolla, CA, United States).

## 3 Results

### 3.1 Pharmacokinetics and tissue distribution

#### 3.1.1 Pharmacokinetics of LN002 in rat plasma content

**FIGURE 2:**
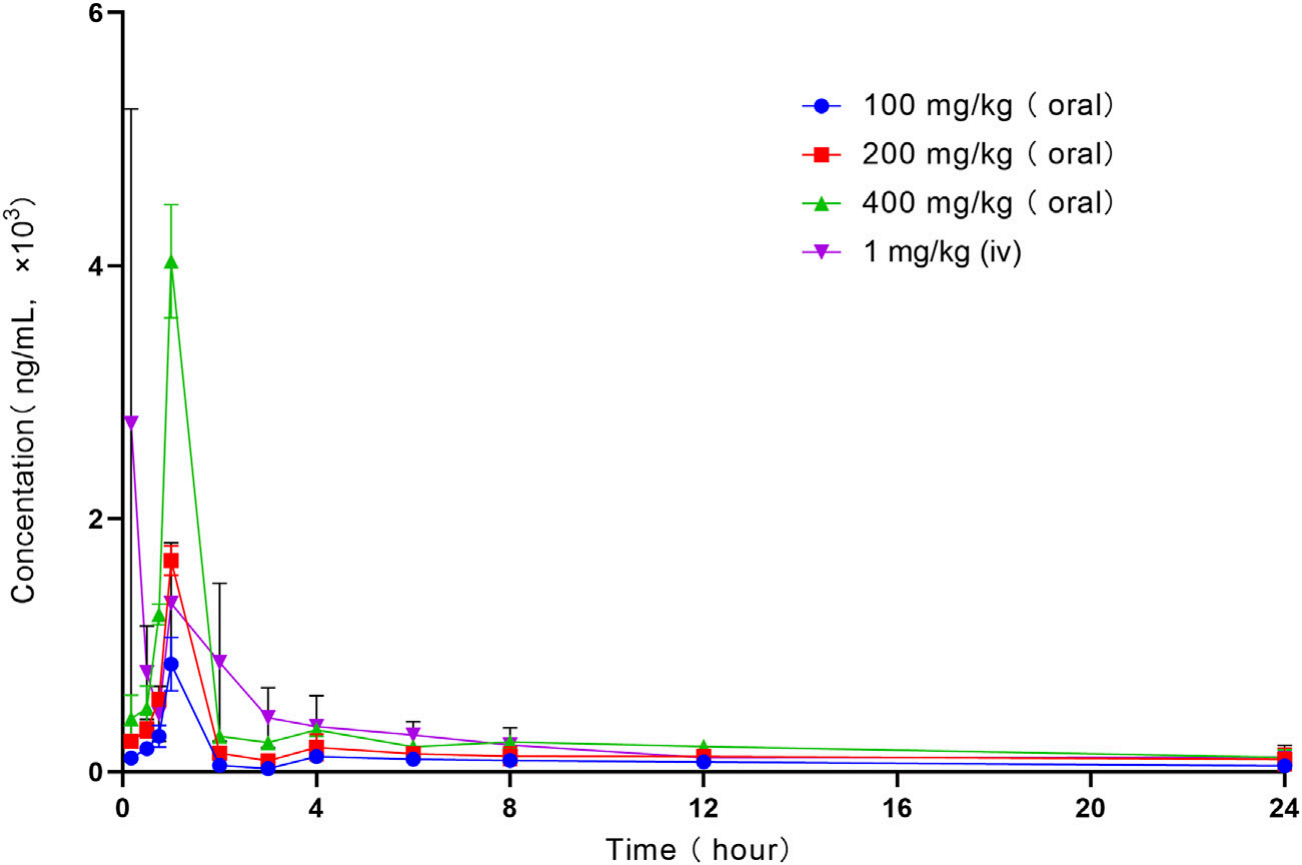
Profile of mean plasma concentration *versus* time following intravenous administration of 1 mg/kg and oral administration of 100, 200, and 400 mg/kg LN002 to rats (n = 6). Oral: oral gavage. iv: intravenous injection. n: number of samples.

The mean plasma concentration–time curve of LN002 in rats after intravenous injection of 1 mg/kg LN002 and oral administration of 100, 200, and 400 mg/kg LN002 is shown in Figure 2, and the PK parameters are shown in Table 1. After intravenous administration of 1 mg/kg LN002, T1/2, AUC0-24 h, AUC0-∞, Cl, and Vd values were 10.91 ± 6.56 h, 7024.86 ± 1,521.51 h⋅ng/mL, 8650.08 ± 1940.68 h⋅ng/mL, 0.11 ± 0.03 L/h/kg, and 1.69 ± 0.44 L/kg, respectively. After oral administration at 100, 200, and 400 mg/kg, Cmax was 849.88 ± 190.02, 1,669.24 ± 105.76, and 4033.21 ± 409.96 ng/mL, respectively. T1/2 was 18.03 ± 5.82, 17.96 ± 14.83, and 18.83 ± 11.36 h, respectively. AUC0-24 h was 2280.41 ± 227.40, 4071.31 ± 778.73, and 7498.10 ± 507.34 h⋅ng/mL, respectively. AUC0-∞ was estimated to be 3530.78 ± 733.56, 6026.26 ± 2314.45, and 11,424.35 ± 3979.23 h⋅ng/mL, respectively. Cl/F was 25.97 ± 12.37, 31.43 ± 18.15, and 39.00 ± 11.72 L/h/kg, respectively. Vd/F was 581.54 ± 270.42, 763.53 ± 314.26, and 869.21 ± 279.40 L/kg, respectively.

**TABLE 1:** Pharmacokinetic parameters of LN002 in plasma following single intravenous injection and gavage in rats (n = 6, mean ± SD).

Mode of administration	Dose (mg/kg)	Plasma						
		T_max_ (h)	C_max_ (ng/mL)	AUC_0–24 h_ (h⋅ng/mL)	T_1/2_ (h)	AUC_0-∞_ (h⋅ng/mL)	V_d_ (L/kg)	Cl (L/h/kg)
Oral	100	1.00	849.88 ± 190.02	2280.41 ± 227.40	18.03 ± 5.82	3530.78 ± 733.56	581.54 ± 270.42	25.97 ± 12.37
	200	1.00	1669.24 ± 105.76	4071.31 ± 778.73	17.96 ± 14.83	6026.26 ± 2314.45	763.53 ± 314.26	31.43 ± 18.15
	400	1.00	4033.21 ± 409.96	7498.10 ± 507.34	18.83 ± 11.36	11,424.35 ± 3979.23	869.21 ± 279.40	39.00 ± 11.72
i.v	1	0.75	2944.39 ± 2162.79	7024.86 ± 1521.51	10.91 ± 6.56	8650.08 ± 1940.68	1.69 ± 0.44	0.11 ± 0.03

SD: standard deviation.

n: number of samples.

T_max_, time of maximum observed concentration.

C_max_, maximum concentration.

AUC_0–24 h_, the area under the concentration–time curve from 0 h to 24 h the sample time point.

T_1/2_, half-life.

AUC_0-∞_, the area under the concentration–time curve from 0 h to the elimination of all original drugs.

V_d_, volume of distribution.

Cl, clearance.

#### 3.1.2 Tissue distribution

**FIGURE 3:**
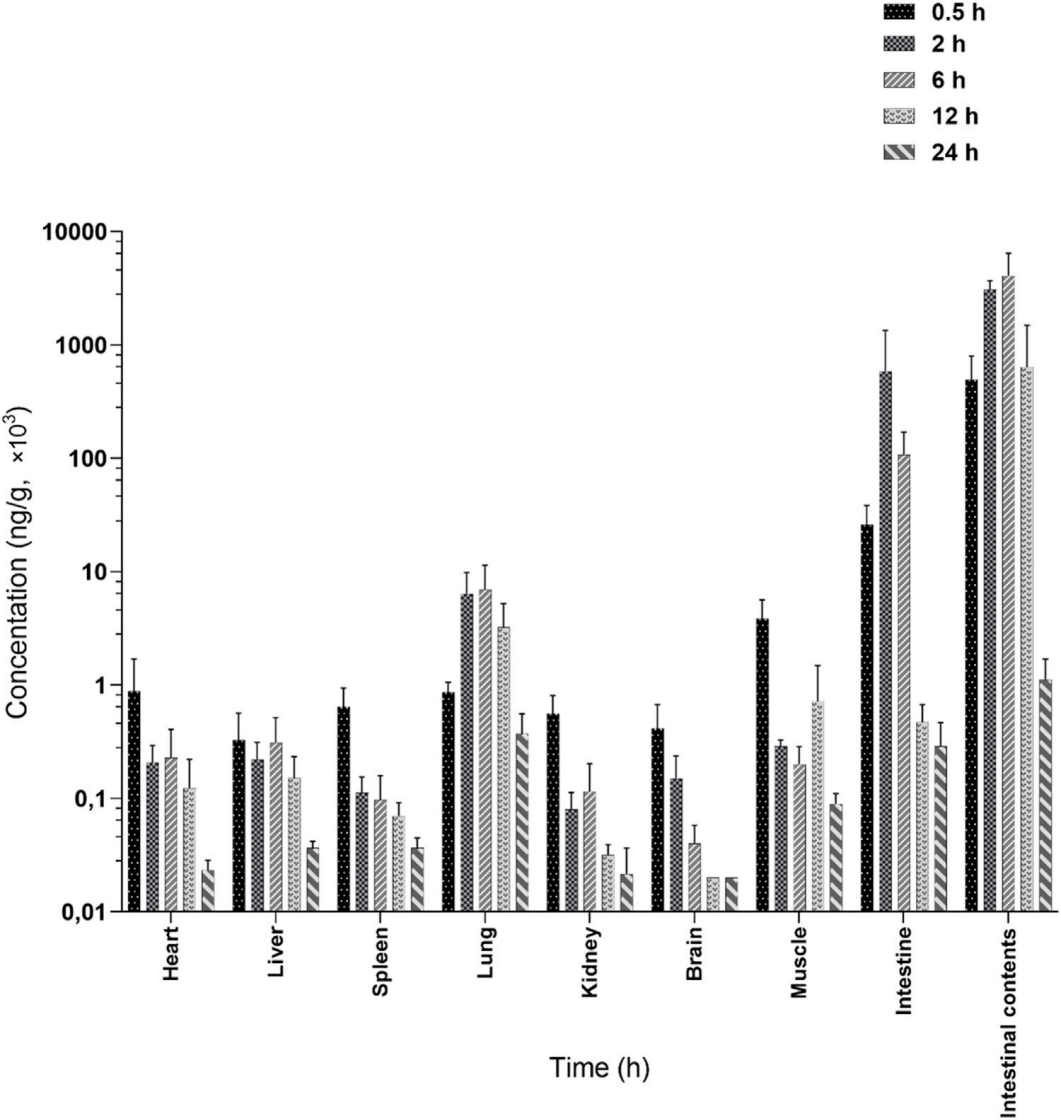
Tissue distribution of LN002 after oral administration of 200 mg/kg to rats (n = 6) (Marzook and Sateriale, 2020; Schubert et al., 2023). n: number of samples.

The tissue and intestinal content distribution results following oral administration of 200 mg/kg LN002 are presented in Figure 3 and Table 2. After oral administration at 200 mg/kg, LN002 was distributed rapidly and extensively. The peak concentration was rapidly reached at 0.5 h and decreased after 2 h, except for the small intestine, intestinal contents, and lungs (2 h). LN002 exhibited its highest concentration in the intestinal contents, followed by the small intestine, lung, muscle, heart, liver, spleen, kidney, and brain.

**TABLE 2:** The main pharmacokinetic parameters of tissues after oral administration of LN002 (200 mg/kg) in SD rats ((n = 6, mean ± SD).

Parameter	Tmax (h)	Cmax (ng/g)	AUC_0-24_ h (h⋅ng/g)	T1/2 (h)
Heart	0.57 ± 0.11	972.31 ± 165.32	3855.84 ± 323.04	6.82 ± 1.65
Liver	0.67 ± 0.19	477.89 ± 157.31	4051.62 ± 586.44	5.54 ± 0.66
Spleen	0.50	645.18 ± 187.03	2280.55 ± 418.06	5.80 ± 1.93
Lung	2.09 ± 0.43	8696.49 ± 1412.58	84,344.09 ± 9677.76	5.43 ± 1.60
Kidney	0.5	560.64 ± 135.29	1781.24 ± 453.85	5.03 ± 1.52
Brain	0.58 ± 0.19	455.86 ± 80.88	1328.60 ± 176.19	5.20 ± 2.31
Muscle	0.58 ± 0.19	3980.60 ± 588.82	12,634.31 ± 2366.24	6.30 ± 2.71
Intestine	2.67 ± 1.49	607,466.00 ± 76,376.49	2185750.70 ± 149,399.23	5.48 ± 1.48
Intestinal contents	3.33 ± 1.60	4452838.33 ± 671,971.29	35080673.50 ± 644,065.57	1.64 ± 0.01

SD: standard deviation.

n: number of samples.

Tmax, time of maximum observed concentration.

Cmax, maximum concentration.

AUC_0–24 h_, the area under the concentration–time curve from 0 h to 24 h the sample time point.

T1/2, half-life.

### 3.2 Sample preparation

In this study, the liver, small intestine, and intestinal contents were selected as quintessential samples for analysis. An optimal extraction solvent was screened by conducting a methodical comparison of the efficacy of these solvents through the metrics of recovery rates and extract purity. The results indicated that the recovery of acetonitrile: methanol (2:1, v/v) was the highest, followed by the recovery of 2% acetonitrile formate and acetonitrile (Table 3). Given that LN002 represents a novel compound, the current body of literature provides no pertinent discussions or analyses relating to its properties or potential applications. In the present investigation, a suite of SPE cartridges, including Poly-Sery MAX SPE, Poly-Sery MCX SPE, SPE-HLB, and Bond Elut C18 (Shanghai Anpu Experimental Technology Co., Ltd., Shanghai, China), were used to ascertain optimal purification conditions. This determination hinged upon comparative analysis of recovery rates and the efficacy of purification. The results are shown in Table 4.

**TABLE 3:** Recovery of LN002 from the liver, small intestine, and intestinal contents extracted from various extracts (n = 3).

Type of extractant	Average recovery (%, mean ± SD)		
	Liver	Intestinal contents	Small intestine
acetonitrile	79.95 ± 3.32	79.96 ± 4.87	88.19 ± 8.47
methanol	88.16 ± 4.14	79.72 ± 4.72	80.27 ± 4.53
ethyl acetate	20.70 ± 0.89	33.73 ± 3.57	16.87 ± 1.63
2% acetonitrile formate	92.61 ± 1.13	91.55 ± 3.82	90.19 ± 4.14
acetonitrile: methanol (2:1, V/V)	97.85 ± 1.42	93.61 ± 3.08	97.93 ± 2.28

SD: standard deviation.

n: number of samples.

**TABLE 4:** Comparison of the purification effects of liquid–liquid extraction and solid-phase extraction (n = 3).

Purification method		Average recovery (%, mean ± SD)		
		Liver	Intestinal contents	Small intestine
Liquid-liquid extraction		75.89 ± 1.57	64.20 ± 2.84	76.69 ± 1.54
Solid phase extraction	MCX	30.79 ± 1.85	18.93 ± 0.82	21.36 ± 0.54
	MAX	93.44 ± 3.25	90.20 ± 1.17	93.65 ± 1.48
	HLB	86.27 ± 1.64	76.10 ± 2.76	85.41 ± 1.21
	C18	18.45 ± 0.56	34.53 ± 11.10	30.57 ± 1.47

SD: standard deviation.

n: number of samples.

### 3.3 Method validation

#### 3.3.1 Specificity

**FIGURE 4:**
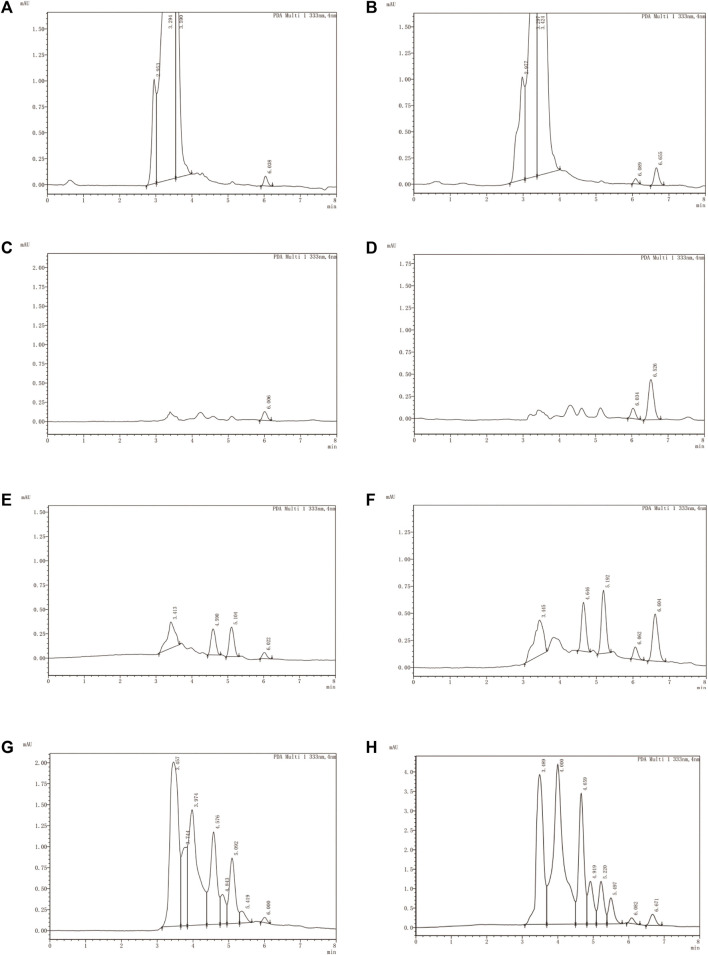
Typical chromatograms of LN002. **(A)** Blank plasma, **(B)** blank plasma spiked with 1,000 ng/mL LN002, **(C)** blank liver, **(D)** blank liver spiked with 1,000 ng/mL LN002, **(E)** small intestine, **(F)** small intestine spiked with 1,000 ng/mL LN002, **(G)** blank intestinal contents, and **(H)** blank intestinal contents spiked with 1,000 ng/mL LN002.

The analytical methodology deployed for quantifying LN002 demonstrated excellent specificity, as evidenced by the absence of pronounced interference from endogenous components within the detected peaks. The retention time was about 6.60 min for LN002. Typical chromatograms were obtained from blank plasma, blank tissues, and intestinal contents spiked with LN002 (Figure 4).

#### 3.3.2 Sensitivity and linearity

The LOD and LOQ of plasma were 10 and 20 ng/mL, respectively. The LOD and LOQ of tissue and intestinal contents were 20 and 50 ng/g, respectively. The calibration ranges, calibration curves, and correlation coefficients of the analytes in each biological sample are included in Table 5. All of the correlation coefficients (*R* ^*2*^) were above 0.99. The sensitivity and accuracy of this analytical method were adequate for application in PK and tissue distribution studies of LN002 in rats.

**TABLE 5:** Calibration curves and calibration ranges for standard operating fluids.

Sample	Calibration range (ng/mL or ng/g)	Calibration curve	R^2^
Plasma	20–1,000 μg/mL	Y = 4951.94X + 0.9792	0.9983
Heart	50–2000	Y = 4948.91X - 3.22128	0.9971
Liver		Y = 21,972.3X + 55.1580	0.9984
Spleen		Y = 4312.16X + 132.855	0.9961
Lung		Y = 4962.83X + 14.3897	0.9976
Kidney		Y = 4962.62X + 16.7457	0.9989
Brain		Y = 5062.83X - 54.7761	0.9958
Muscle		Y = 4565.63X + 1.21434	0.9999
Intestine		Y = 5363.85X + 107.514	0.9978
Intestinal contents		Y = 21,943.1X + 7.83820	0.9997

#### 3.3.3 Accuracy and precision

The analytical fidelity of LN002, evaluated through intra-day and inter-day assays, yielded results that adhered to the acceptable thresholds of accuracy and precision in various matrices including plasma, tissue homogenate, and intestinal contents. This consistent performance underscores the reliability of LN002 as a robust analytical compound in diverse biological specimens. The relevant values are listed in Table 6. The intra-day accuracy ranged from 76.30% to 104.89%; the intra-day CV ranged from 0.84% to 7.70%, and the inter-day CV ranged from 2.14% to 6.95%. These data showed that the established methodology was robust and reproducible.

**TABLE 6:** Accuracy and precision of LN002 in rat plasma (six replicates per day for 3 days).

Sample	Nominal concentration (ng/mL or ng/g)	Intra-day						Inter-day
		Day 1		Day 2		Day 3		
		Average recovery (%, mean ± SD)	Correlation of variation (%, CV, n = 6)	Average recovery (%, mean ± SD)	Correlation of variation (%, CV, n = 6)	Average recovery (%, mean ± SD)	Correlation of variation (%, CV, n = 6)	Correlation of variation (%, CV, n = 18)
Plasma	20	98.90 ± 2.00	2.20	98.47 ± 3.81	3.87	95.88 ± 4.55	4.75	3.75
	50	94.84 ± 4.66	4.91	94.28 ± 4.42	4.69	92.55 ± 4.16	4.50	4.55
	200	97.67 ± 2.94	3.01	99.25 ± 1.28	1.29	95.29 ± 1.48	1.56	2.61
	1,000	101.37 ± 2.78	2.74	103.14 ± 1.09	1.05	101.94 ± 2.34	2.29	2.14
Heart	50	90.08 ± 4.38	4.87	91.04 ± 3.17	3.48	91.16 ± 2.53	2.77	3.61
	200	91.94 ± 2.99	0.84	93.11 ± 0.78	4.28	87.22 ± 3.74	1.99	4.09
	1,000	88.20 ± 1.76	1.99	88.94 ± 0.60	0.68	88.67 ± 5.12	5.77	3.35
Liver	50	82.07 ± 4.85	5.91	86.56 ± 4.02	4.64	83.23 ± 2.29	2.75	4.92
	200	81.94 ± 1.82	2.22	85.87 ± 1.42	1.65	86.31 ± 1.88	2.18	3.06
	1,000	80.21 ± 3.98	4.97	93.14 ± 2.22	2.38	86.91 ± 1.56	1.80	6.95
Spleen	50	88.60 ± 2.10	2.37	90.89 ± 1.88	2.07	88.61 ± 3.86	4.36	3.16
	200	90.24 ± 1.65	1.82	86.14 ± 1.90	2.21	91.40 ± 1.07	1.17	3.09
	1,000	90.73 ± 3.13	3.44	90.50 ± 1.57	1.74	90.25 ± 1.85	2.05	2.38
Lung	50	89.32 ± 5.26	5.87	89.49 ± 3.28	3.67	89.89 ± 1.33	1.47	3.84
	200	89.73 ± 4.84	5.39	91.84 ± 2.02	2.20	89.02 ± 1.80	2.02	3.60
	1,000	90.78 ± 4.53	4.99	92.68 ± 2.01	2.17	90.81 ± 1.87	2.06	3.30
Kidney	50	88.44 ± 5.61	6.34	89.96 ± 4.11	4.56	91.25 ± 2.37	2.60	4.62
	200	88.89 ± 3.95	4.44	90.28 ± 2.25	2.50	90.37 ± 2.48	2.75	3.22
	1,000	86.46 ± 2.47	2.85	90.74 ± 2.11	2.33	89.25 ± 1.79	2.00	4.06
Brain	50	88.85 ± 4.43	4.99	88.35 ± 3.45	3.91	89.05 ± 2.94	3.30	3.89
	200	91.21 ± 3.80	4.17	89.44 ± 2.57	2.87	92.39 ± 1.57	1.69	3.20
	1,000	93.96 ± 2.99	3.18	92.54 ± 1.54	1.67	90.20 ± 3.24	3.59	3.24
Muscle	50	92.50 ± 1.81	1.96	90.22 ± 1.78	1.97	87.72 ± 5.77	6.58	4.40
	200	90.79 ± 3.02	3.33	87.27 ± 1.57	1.80	94.18 ± 1.60	1.70	3.91
	1,000	90.31 ± 2.64	2.92	90.03 ± 3.73	4.14	91.54 ± 4.76	5.20	4.02
Intestine	50	89.00 ± 6.85	7.70	88.40 ± 4.21	4.76	89.06 ± 5.24	5.88	5.87
	200	88.69 ± 4.09	4.61	89.58 ± 3.34	3.73	89.34 ± 3.52	3.94	3.88
	1,000	85.73 ± 2.45	2.85	91.40 ± 2.41	2.63	92.84 ± 1.98	2.14	4.25
Intestinal contents	50	92.15 ± 4.89	5.31	89.57 ± 3.53	3.94	91.31 ± 4.07	4.45	4.50
	200	92.94 ± 3.93	4.23	92.90 ± 3.26	3.51	91.88 ± 2.28	2.48	3.32
	1,000	90.12 ± 1.61	1.78	88.50 ± 2.44	2.75	89.81 ± 3.09	3.44	2.70

SD: standard deviation.

CV: coefficient of variability.

n: number of samples.

#### 3.3.4 Stability

Table 7 summarizes all investigated stability data for the analytes in rat plasma, liver, and intestinal contents. Table 8 presents the stability data of the stock solution, indicating that LN002 had no appreciable degradation under the previously mentioned conditions. LN002 was stable in plasma and tissue homogenate.

**TABLE 7:** Stability of LN002 in plasma, tissue homogenate, and intestinal contents (n = 6).

Sample	Nominal concentration (ng/mL or ng/g)	Short-term stability at room temperature (6 h)		Freeze and thaw stability at −20°C/Room temperature (3 cycles)		Auto-sampler stability (12 h)		Long-term stability at −20°C (30 days)	
		Measured concentration (mean ± SD)	Correlation of variation (%, CV)	Measured concentration (mean ± SD)	Correlation of variation (%, CV)	Measured concentration (mean ± SD)	Correlation of variation (%, CV)	Measured concentration (mean ± SD)	Correlation of variation (%, CV)
Plasma	50	0.051 ± 0.003	6.47	0.048 ± 0.002	4.03	0.050 ± 0.002	4.10	0.052 ± 0.002	4.00
	200	0.210 ± 0.013	6.40	0.197 ± 0.014	7.24	0.204 ± 0.014	6.76	0.203 ± 0.006	3.04
	1,000	0.987 ± 0.032	3.29	1.028 ± 0.035	3.38	0.992 ± 0.061	6.15	1.035 ± 0.080	7.69
Liver	50	0.050 ± 0.003	5.56	0.051 ± 0.002	3.25	0.049 ± 0.002	4.63	0.051 ± 0.002	4.11
	200	0.197 ± 0.009	4.76	0.205 ± 0.005	2.61	0.198 ± 0.007	3.32	0.196 ± 0.007	3.66
	1,000	0.984 ± 0.042	4.27	0.985 ± 0.057	5.80	1.065 ± 0.036	3.35	0.995 ± 0.064	6.70
Intestinal contents	50	0.051 ± 0.002	3.37	0.050 ± 0.002	4.19	0.048 ± 0.001	3.08	0.048 ± 0.002	2.14
	200	0.204 ± 0.007	3.42	0.192 ± 0.009	4.88	0.191 ± 0.011	5.77	0.202 ± 0.003	1.29
	1,000	0.976 ± 0.042	4.27	0.978 ± 0.053	5.44	1.001 ± 0.034	3.44	0.997 ± 0.012	1.16

SD: standard deviation.

CV: coefficient of variability.

n: number of samples.

**TABLE 8:** Stability of LN002 stock solution (n = 6).

Analytes	Nominal concentration (ng/mL)	15 d		30 d	
		Measured concentration (mean ± SD)	Correlation of variation (%, CV)	Measured concentration (mean ± SD)	Correlation of variation (%, CV)
LN002	1,000	0.941 ± 0.091	9.62	0.924 ± 0.051	5.47

SD: standard deviation.

CV: coefficient of variability.

n: number of samples.

## 4 Discussion

The mean plasma concentration–time curve of LN002 indicates that the second peak in blood concentration approximately 1 h after intravenous injection can be ascribed to the properties of the drug. Despite its high insolubility in water, the compound demonstrates favorable lipophilicity. Upon intravenous administration, the drug undergoes rapid distribution into tissues, followed by re-release into the bloodstream, resulting in the emergence of the second peak. After oral administration of LN002 in rats, the peak concentration in plasma reached 1 h. LN002 was eliminated slowly after oral administration, and evident accumulation was found with mean residence time. Moreover, the PK process in rats was linear. This result indicates that this molecule follows linear PK behavior. The low oral administration F values, 0.32% with 100 mg/kg, 0.29% with 200 mg/kg, and 0.27% with 400 mg/kg LN002, may be related to the large estimated V_d_ and Cl values. LN002 exhibited poor total exposure in rats after oral administration at 100, 200, and 400 mg/kg. As *Cryptosporidium* chiefly targets the intestinal epithelium, systemic exposure may not be required for efficacy, concurrently amplifying its safety profile by limiting systemic exposure. Comprehensive preclinical safety and pharmacological preclinical assessments are necessary to support the progression toward animal’s clinical trials.

The results of the tissue distribution study of LN002 indicating that LN002 had a high affinity for the intestine. As the structure of this compound is a conjugated system, its structural properties indicate that this compound is difficult to concentrate in water. After oral administration of LN002, it directly enters the intestine, so the concentration in the intestinal contents is high, followed by the small intestine. The epithelium of the small intestine is the main site of *Cryptosporidium* infection, so the exposure of LN002 in the small intestine is more effective for therapeutic purposes. In addition, higher concentrations of LN002 in the lungs may be related to experimental operations. LN002 could be detected at 24 h in all tissues and intestinal contents, showing evident accumulation in any of the tested tissues. The concentration of LN002 in various tissues decreased rapidly after reaching the peak concentration, which was in accordance with the trend observed in plasma.

The use of protein precipitation (PPT) with acetonitrile as the solvent is a common method for preparing plasma samples (Alshogran et al., 2023; Zhang et al., 2024). Although methanol is often used for PPT, it was found to result in poor extraction recovery compared with acetonitrile. Consequently, the decision was made to use acetonitrile as the precipitation solvent for plasma sample preparation because of its high extraction recovery, meeting the necessary criteria for analysis. This approach has been widely adopted for the extraction of biological samples, ensuring accurate and reliable results. An exhaustive examination of extant scholarly texts reveals that a plethora of extraction solvents have been used in procuring substances from tissue specimens, including but not limited to acetonitrile (Toro-Cordova et al., 2016; Song et al., 2021b; Yoshioka et al., 2022), methanol (Das et al., 2023; Ozukum et al., 2023), ethyl acetate (Marefati et al., 2022), 2% acetonitrile formate (Sun et al., 2023; Wen et al., 2023), and acetonitrile: methanol (2:1, v/v) (Coughlin et al., 2011). The study’s findings indicate that the recovery of 2% acetonitrile formate, acetonitrile, and methanol is high, but it is not suitable as an extract because of the high price and toxicity of acetonitrile, as well as the relatively large number of impurities extracted from methanol. The recovery of ethyl acetate after extraction is low, and it cannot meet the analytical requirements. Acetonitrile:methanol (2:1, v/v) has a good extraction effect, few impurities, and little influence on the analysis results; thus, acetonitrile:methanol (2:1, v/v) was selected as the extract for the extraction solvent of LN002 from rat tissues and intestinal contents. In obtaining a better purification effect while minimizing the loss of compounds, the tissue homogenate (liver and small intestine) and intestinal contents were purified using two different methods, namely, liquid–liquid extraction (LLE) and solid extraction (Sun et al., 2023). The results showed that the Poly-Sery MAX SPE Cartridge emerged as superior, yielding high levels of analyte recovery, impressive purification performance, and minimal impurity intrusion. Consequently, the Poly-Sery MAX SPE Cartridge was selected as the extraction column for this experiment.

This study developed and validated an HPLC method quantitation for LN002 within plasma, tissues, and intestinal content specimens. This method has been used in the execution of PK explorations, assessment of absolute bioavailability, and tissue distribution of LN002 in rats. After oral administration, LN002 exhibited low absolute oral bioavailability, and it is characterized by prompt systemic uptake juxtaposed with a protracted clearance phase. Apart from its rapid and extensive distribution, LN002 was highly present in the small intestine, indicating its potential as an effective anti-*Cryptosporidium* compound. Consequently, this study provides valuable insights into future e investigations on LN002. In this study, we studied the absorption and distribution of LN002 in rats, which had certain limitations to evaluate the efficacy and safety of the drug. Further research on the metabolism and excretion of LN002 in rats is needed to provide strong support for rational drug use, drug development and clinical application.

## Data Availability

The raw data supporting the conclusions of this article will be made available by the authors, without undue reservation.
