# Supplementary figures and images for: Pharmacokinetics and tissue distribution of LN002, a new compound alternative oxidase inhibitor against Cryptosporidium in rats
Source: Front Pharmacol. 2024 Jul 30;15:1413872. doi: 10.3389/fphar.2024.1413872 (PMC11325084; doi:10.3389/fphar.2024.1413872)

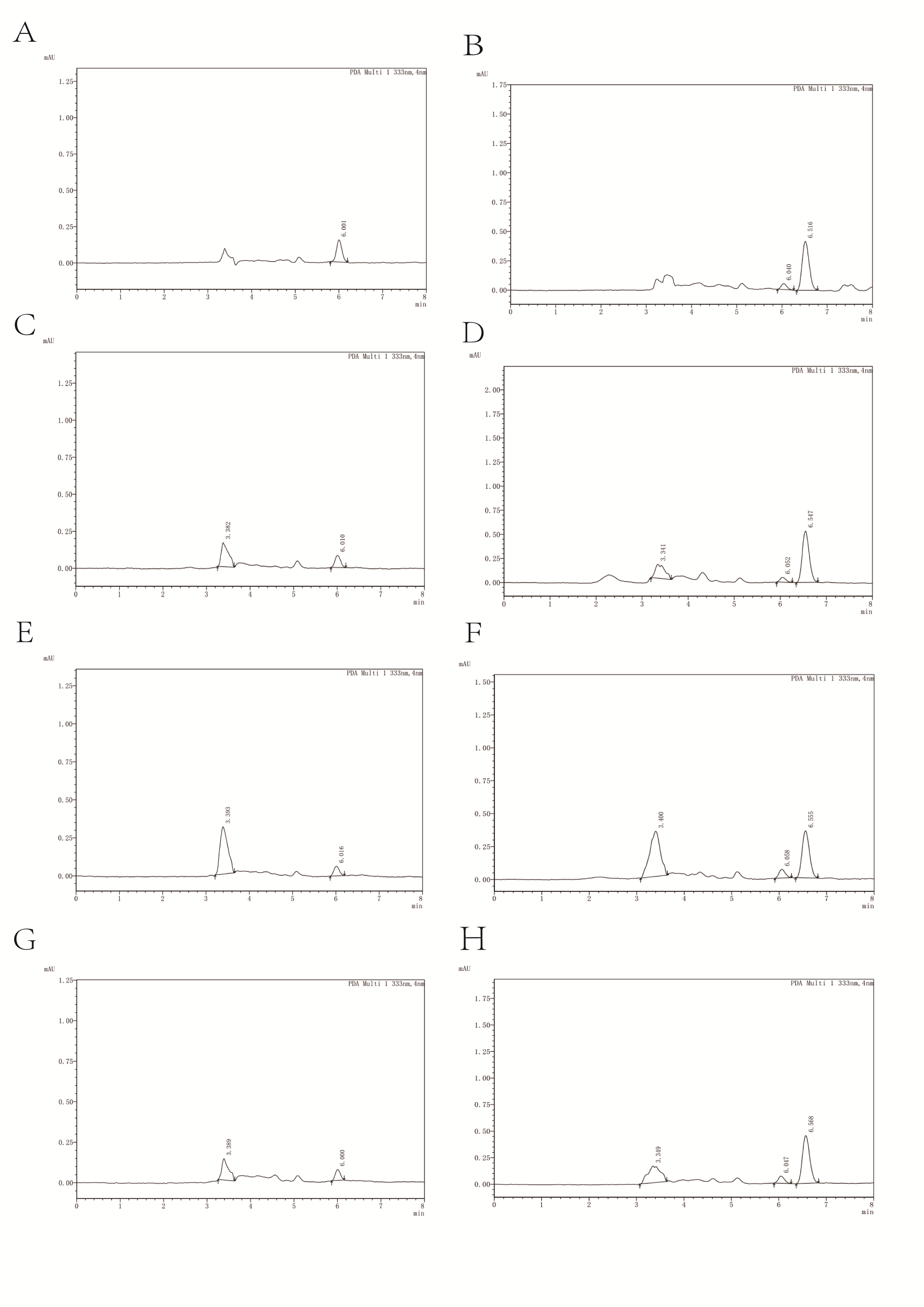

Supplement: Supplementary file 1 [file DataSheet1.zip › Figure S1 (A-H).TIF]

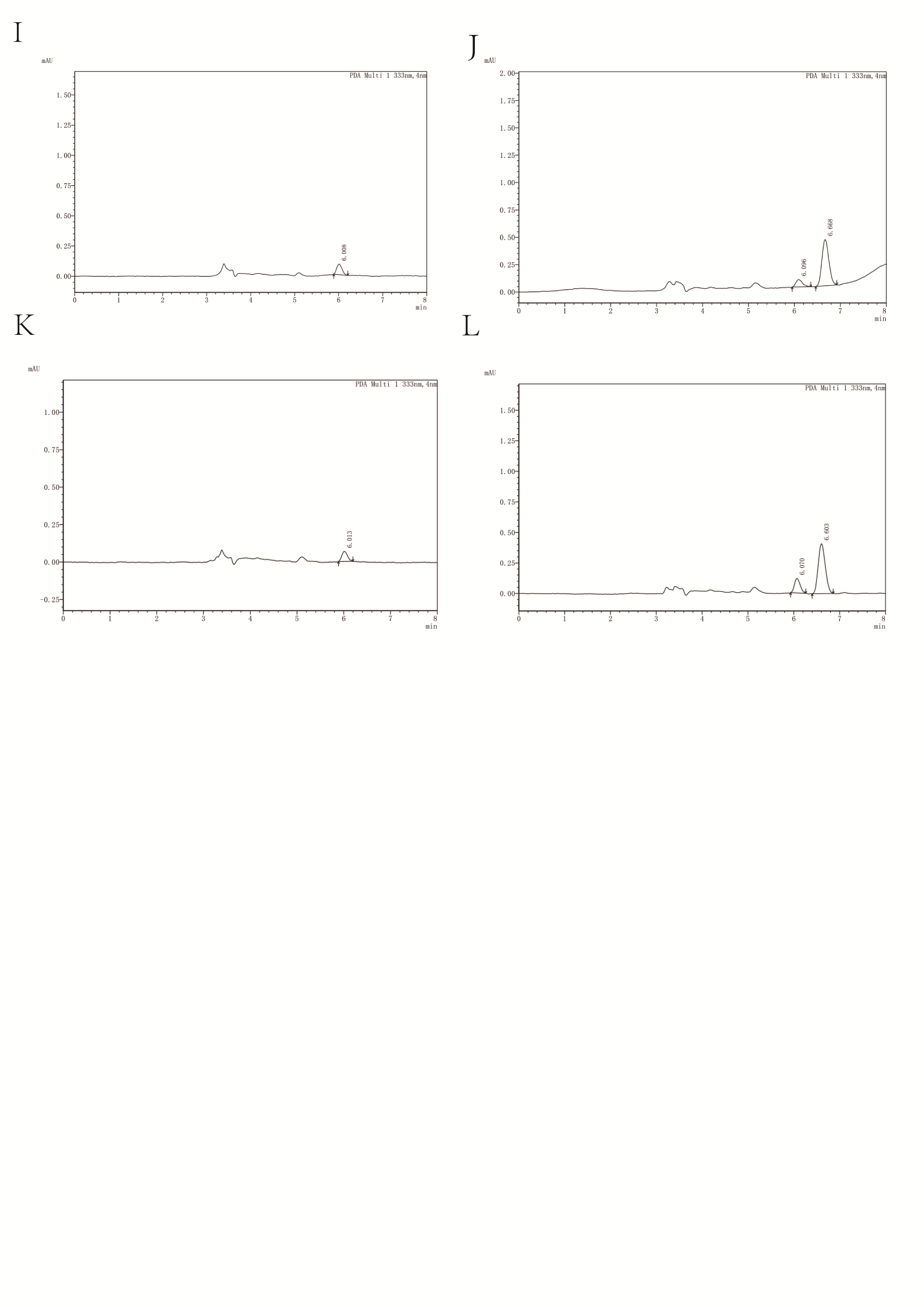

Supplement: Supplementary file 1 [file DataSheet1.zip › Figure S1 (I-L).TIF]
